# Introductory paragraph

**DOI:** 10.1177/0269215520948753

**Published:** 2020-08-03

An unusual issue: short, and all the papers are evaluative: one randomized trial, five systematic reviews. Also one paper is unusually long. When I was a very junior doctor, I was taught (by physiotherapists) that resistance training was very bad for patients after stroke. Now a systematic review found 30 trials (1051 patients) evaluating it. It does not seem harmful, and in some respects is equal to other therapies. There was no evidence of superiority to other therapies. I am still uncertain why anyone tried extra-corporeal shockwave therapy for spasticity, but a systematic review shows it reduces spasticity in the arm is people late after stroke. Practical as a treatment? Shoulder pain attributed to rotator-cuff disorder was the subject of the third systematic review. Does resisted or non-resisted exercise help? Resisted exercises might, but as usual the evidence base is not strong. The fourth review is a scoping review of interventions to reduce sedentary behaviour or increase activity (not the same thing) in hospital inpatients. The range of interventions is large; whether any help is uncertain. The last review includes a network metanalysis comparing different pairs of treatments for trigger finger. Surgery was the best bet in the longterm. The randomized controlled trial studies many patients (n = 150), and compared five different bandages for arm lymphoedema after breast cancer treatment. There were difference but with only 30 in each group it was difficult to know whether any was actually worse.

